# Comparative Efficacy and Safety of Moxifloxacin and Levofloxacin in a short standardised Rifampicin Resistant TB regimen: A STREAM 2 secondary analysis

**DOI:** 10.3390/tropicalmed9090211

**Published:** 2024-09-11

**Authors:** Stella M. Fabiane, Chen-Yuang Chiang, Sarah K. Meredith, Meera Gurumurthy, Adamu Bayissa, Andrew J. Nunn, Ruth L. Goodall, on behalf of the STREAM Collaborators, AK Bhatnagar, F Conradie, N Gopalan, Bruce Kirenga, N Kiria, D Meressa, R Moodliar, N Ngubane, M Rassool, R Solanki, B Tsogt, E Tudor, G Torrea

**Affiliations:** Rajan Babu Institute for Pulmonary Medicine & Tuberculosis (RBIPMT), Delhi, India; Empilweni TB Hospital, Eastern Cape, South Africa; ICMR-National Institute for Research in Tuberculosis, Chennai, India; https://ror.org/03dmz0111 Makerere University Lung Institute, https://ror.org/02rhp5f96 Mulago Hospital, Kampala, Uganda; https://ror.org/02kf03x09 National Center for Tuberculosis and Lung Diseases, Tbilisi, Georgia; St. Peter’s Tuberculosis Specialized Hospital and Global Health Committee, Addis Ababa, Ethiopia; THINK: Tuberculosis & HIV Investigative, Doris Goodwin Hospital, Pietermaritzburg, South Africa; King Dinuzulu Hospital Complex, Durban, South Africa; Clinical HIV Research Unit, https://ror.org/04nrs5002 Helen Joseph Hospital, Department of Internal Medicine, https://ror.org/03rp50x72 University of the Witwatersrand, Johannesburg, South Africa; https://ror.org/0408b4j80 B.J. Medical College, Ahmedabad, India; Mongolian Anti-Tuberculosis Coalition, Ulaanbaatar, Mongolia; https://ror.org/02j0at714 Institute of Phthisiopneumology “Chiril Draganiuc”, Chisinau, Republic of Moldova; Institute of Tropical Medicine, Antwerp, Belgium; 1 https://ror.org/001mm6w73 MRC Clinical Trials Unit at University College London, 90 High Holborn, London WC1V 6LJ, UK; 2 College of Medicine, https://ror.org/05031qk94 Taipei Medical University, Taiwan; 3 https://ror.org/05mdyn772 Vital Strategies, Singapore; 4 https://ror.org/05mfff588 Armauer Hansen Research Institute (AHRI), Addis Ababa, Ethiopia

**Keywords:** moxifloxacin, levofloxacin, efficacy treatment outcomes, safety treatment outcomes

## Abstract

**Background:**

The World Health Organisation (WHO) categorises moxifloxacin and levofloxacin as Group A drugs which should be prioritised in the treatment of rifampicin-resistant tuberculosis. We compare their relative efficacy and safety using data from the STREAM trial

**Methods:**

Marginal structural models were used to balance differences in the baseline characteristics of participants receiving the STREAM control regimen containing either moxifloxacin or levofloxacin as this was not a randomised comparison. The difference in proportions between regimens was estimated for favourable outcome, any grade 3/4 adverse event, QTcF increase to ≥500msec, QTcF increase from baseline by at least 60 msec, and any grade 3/4 adverse event excluding QT events, using weighted analyses

**Results:**

In efficacy analyses (n=123) the weighted risk difference (moxifloxacin – levofloxacin, wRD) for a favourable outcome was -0.045 (-0.213, 0.123), p=0.60. Similarly, estimates from the safety analyses (n=127) showed no evidence of a difference between the fluoroquinolones, other than a suggestion of fewer QTcF increases from baseline on levofloxacin (wRD 0.160 (-0.026, 0.346), p=0.091)

**Conclusions:**

In this small dataset we found no statistically significant difference in key efficacy or safety outcomes between the moxifloxacin- and levofloxacin-containing regimens; there was a suggestion that QTcF increases from baseline were fewer on levofloxacin.

## 1 Introduction

Fluoroquinolones were first investigated and found to be effective for the treatment of rifampicin-resistant TB (RR-TB) over 30 years ago [1]. In 2010 Van Deun et al reported on the results of six cohort studies conducted in Bangladesh which evaluated the effectiveness of standardized regimens for patients with proven multidrug-resistant tuberculosis (MDR-TB). The most promising results were obtained from a regimen containing high-dose gatifloxacin, a fourth-generation fluoroquinolone, clofazimine, ethambutol and pyrazinamide supplemented by prothionamide, kanamycin, and high-dose isoniazid during an intensive phase of a minimum of 4 months [2]. The STREAM (Standardised Treatment Regimen of Anti-TB Drugs for Patients with MDR-TB) trial Stage 1 was designed to assess whether the 9-month regimen developed in Bangladesh, was as effective as the WHO regimen of 20 or more months recommended at the time [3, 4]. Gatifloxacin was replaced by high dose moxifloxacin because 1) quality assured gatifloxacin was no longer available, 2) moxifloxacin was considered as efficacious as gatifloxacin [5] and 3) moxifloxacin was superior to levofloxacin in an animal study [6].

Stage 2 of STREAM was designed before the results of Stage 1 were known, the objective was to assess whether a fully oral 9-month regimen, in which bedaquiline was substituted for the injectable kanamycin, was as effective as the 9-month regimen studied in Stage 1. The results of Stage 1 indicated that many patients on the 9-month regimen experienced QT prolongation. This led to an amendment to the Stage 2 protocol in which levofloxacin was substituted for moxifloxacin in the Stage 2 control regimen, with the aim of reducing the frequency of QT prolongation [7], as levofloxacin was thought to have less risk of QT prolongation [8]. As a consequence, some participants on the control regimen were treated with moxifloxacin and some with levofloxacin.

Results presented in the primary analysis of Stage 2 reported findings irrespective of which fluoroquinolone was used in the control regimen, and separately for moxifloxacin and levofloxacin. Overall, the all-oral regimen was shown to be superior in efficacy to the control. When moxifloxacin was used in the control regimen the difference in outcome was 9·2% (95% CI –1·2 to 19·6) when compared to the oral regimen; the corresponding difference when levofloxacin was used was 14·7% (2·5 to 26·8). That is to say, the oral regimen did not achieve statistical superiority to the control regimen with moxifloxacin, whereas it was superior to the control regimen with levofloxacin, raising the possibility that both the efficacy and safety of the two fluoroquinolones might differ. However, no direct comparison between the two fluoroquinolones was made.

Since 2018 WHO has categorised both moxifloxacin and levofloxacin as Group A drugs which should be prioritised in the treatment of RR-TB, but there is no guidance about which to choose or whether they are similar in terms of efficacy and safety. The objective of the present secondary analysis is to assess whether or not there were differences in efficacy and safety outcomes between the moxifloxacin and levofloxacin containing control regimens. Since the choice of fluoroquinolone allocated was not part of a randomised comparison, statistical methods are used to account for potential differences between the populations receiving each drug.

## 2 Methods

STREAM Stage 2 was a randomised, phase 3, non-inferiority trial conducted in 13 clinical sites in 7 countries. The trial methods and primary results at week 76 have been published [6]. In brief, eligible participants were aged 15 years or older (where approved, otherwise 18 years or older) and had pulmonary tuberculosis with evidence of resistance to rifampicin regardless of susceptibility to isoniazid. Participants were ineligible if they were infected with a strain of *Mycobacterium tuberculosis* with evidence of resistance to a second-line injectable drug or fluoroquinolone using line-probe assay.

The Union Ethics Advisory Group was the global ethics committee. Ethical approvals were also obtained from national and institutional ethics committees of participating sites and written informed consent was obtained from all participants.

Participants were randomised to either the Long, Control, Oral or Six-month regimen in the ratio 1:2:2:2, but the primary comparison was between the Control and Oral regimens. The Control was a 9-month regimen that included moxifloxacin (at higher-than-standard dose), clofazimine, ethambutol and pyrazinamide for 40 weeks, with kanamycin, high-dose isoniazid and prothionamide given for the 16-week intensive phase. In the Oral regimen, which was also prescribed for 9-months, bedaquiline was given for 40 weeks, replacing kanamycin and levofloxacin replaced moxifloxacin. In all the regimens the intensive phase could be extended by up to 8 weeks for delayed sputum smear conversion.

Sputum samples for smear and culture were obtained at the randomisation visit, and then every visit from Week 4 up to and including Week 76. The trial reference laboratory tested M. tuberculosis isolates obtained from sputum specimens collected at screening, at randomisation and from Week 8 onwards for phenotypic drug susceptibility and genotyped strains to distinguish true relapses from exogenous reinfections. Regular electrocardiographic (ECG) monitoring with centralised calculation of the corrected QT using Fridericia’s formula (QTcF) was recorded until Week 76.

The outcomes chosen for comparisons between the fluoroquinolones were favourable status at week 76, severe adverse events (classified as adverse events Grade 3 or higher according to the Division of AIDS, National Institute of Allergy and Infectious Diseases [9]), QTcF increase to ≥500msec, and QTcF increase from baseline of at least 60 msec.

A favourable status at 76 weeks was defined as a negative culture for M. tuberculosis at Week 76 and on the preceding visit, with no previous unfavourable outcome. An unfavourable outcome was defined by the initiation of bedaquiline, kanamycin, linezolid or two or more drugs that were not part of the assigned regimen; treatment extension beyond the permitted duration; death from any cause; a positive culture from one of the two most recent specimens, or no Week 76 visit.

## 3 Statistical Methods

The modified intention-to-treat (mITT) population was used for efficacy analyses; it included all randomly assigned participants with a positive culture for *M tuberculosis* at screening or randomisation, except for participants with isolates taken before randomisation who were subsequently found to be susceptible to rifampicin or resistant to both fluoroquinolones and second-line injectables on phenotypic drug-susceptibility testing. The safety population consisted of all randomised participants who received at least one dose of their allocated medication. Participants from India and South Africa were excluded from all analyses reported here since these two countries did not have any participants randomised to the control regimen containing levofloxacin. Analyses of QT prolongation greater than or equal to 500ms were restricted to Mongolia and Uganda as we observed no QT prolongation events in the other countries.

Selected baseline covariates (sex, age, weight, HIV status, QTcF, radiographic lung opacity and cavitation, sputum smear and culture results) were described using frequencies and percentages and were compared between the two different fluoroquinolones using the chi-squared test (all covariates were categorised).

Logistic regression models were used to explore associations between baseline characteristics and each selected outcome. Characteristics found to be associated at the 10% level were then included in a logistic regression along with country, sex, age, BMI, and HIV status (considered a priori to be important) to generate inverse-probability weights; average treatment effect was used for the final weighted logistic regression, excluding those with probabilities of <0.05% or >0.95% and outlier weights. Sensitivity analyses were performed fitting unweighted logistic regressions adjusted for the same covariates present in the weight-generating step. All statistical analyses were carried out using STATA version 17.0.

## 4 Results

In total, 127 participants randomised to the Control regimen were part of the safety analyses population reported here: 62 to a regimen containing levofloxacin and 65 to a regimen containing moxifloxacin. Two-thirds of participants were male, with approximately half having multiple cavities on baseline chest x-ray. There was a suggestion that participants receiving levofloxacin were heavier and were more likely to have a baseline QTcF ≥400msec (Table 1). The efficacy analysis population contained 4 fewer participants: 60 in the levofloxacin-containing regimen and 63 in the moxifloxacin containing-regimen.

In the efficacy analysis (favourable status, Table 2) the weighted odds ratio (wOR) of moxifloxacin relative to levofloxacin was 0.787 (95% CI 0.322, 1.926), with a risk difference (RD) of -0. 045 (95% CI -0.213, 0.123). Estimates from the adjusted analysis similarly showed no statistically significant differences between the performance of the fluoroquinolones: adjusted odds ratio (aOR) 0.526 (95% CI 0.176, 1.571), RD -0.092 (95% CI -0.247, 0.063).

We observed no difference in the number of participants experiencing a grade 3/4 adverse event or a grade 3/4 adverse event excluding QT events between the two fluoroquinolone groups. Although we observed no difference between the groups in QTcF increase to ≥500msec, our data support the possibility that participants on levofloxacin had fewer QTcF increases by at least 60 msec from baseline (Table 3).

The distribution of acquired drug resistance was not indicative of any systematic pattern. Two of four participants who experienced an unfavourable bacteriological outcome on the control regimen including levofloxacin acquired resistance, using phenotypic or gentotypic tests, to kanamycin. Three of five participants on the control regimen including moxifloxacin who experienced an unfavourable bacteriological outcome acquired resistance to clofazimine, fluroquinolones or pyrazinamide only.

## 5 Discussion

Results presented in the primary efficacy analysis of STREAM Stage 2 suggested that whichever fluoroquinolone was used in the control regimen, efficacy outcomes were inferior to those in participants receiving the fully oral regimen, but whether there were any differences between the moxifloxacin- and the levofloxacin-based control regimen was not formally examined. The present analysis in which adjustments have been made for differences in the baseline characteristics between the two versions of the control regimen suggest that there is no difference in the efficacy or safety of the regimen based on which fluoroquinolone it contained, other than a suggestion of a benefit of levofloxacin in terms of fewer increases in QTcF from baseline.

There are limited randomised clinical data available comparing outcomes in RR- or MDR-TB when using either levofloxacin or moxifloxacin. A multicentre open-label trial compared the effectiveness of levofloxacin and moxifloxacin among patients with MDR-TB in South Korea [10]. A total of 151 participants were included in the final analysis, outcomes using WHO 2013 definitions were very similar between the levofloxacin and moxifloxacin groups, cure rates of 83.1 vs. 78.4% respectively, P = 0.54 and treatment success rates 84.4 vs. 79.7% respectively, P = 0.53. Patients in the levofloxacin group had more adverse events than those in the moxifloxacin group (79.2 vs. 63.5%, P = 0.03), especially musculoskeletal ones (37.7 vs. 14.9%, P = 0.001).

An exploratory analysis of treatment outcomes of levofloxacin- and moxifloxacin-based regimens for MDR-TB in an Ethiopian study demonstrated significantly better efficacy outcomes in the levofloxacin-based group. There was no difference in culture conversion rates; adverse events were more frequent in the moxifloxacin-based group [11].

Moxifloxacin and levofloxacin are currently both widely used fourth-generation fluoroquinolones and both are recommended by WHO for use in RR-TB regimens [12], but there may be a better alternative, gatifloxacin. Observational studies have reported that gatifloxacin-based short regimens achieve a high treatment success proportion in the treatment of MDR-TB [13, 14]. The original Bangladesh short regimen, on which the STREAM control regimen was based, used gatifloxacin as it was a cheaper alternative to high-dose moxifloxacin which had been shown to possibly suppress the drug-resistant mutant population more effectively than standard-dose moxifloxacin [4]. A Chinese study reports 83.3% treatment success among MDR-TB patients treated with a gatifloxacin-based short regimen [15].

Although moxifloxacin was selected for the STREAM trial, it was not clear whether moxifloxacin would perform as well as gatifloxacin, but favourable outcome rates for the control regimen in STREAM were high [4]. However, a recent study reported that gatifloxacin was superior to levofloxacin and moxifloxacin in short treatment regimens for MDR-TB. Patients treated with either a levofloxacin-based or moxifloxacin-based regimen had a higher risk of unfavourable outcomes than those treated with gatifloxacin-based regimens [16]. Furthermore, no patient treated with gatifloxacin-based regimens had acquired fluoroquinolone resistance. In contrast, 4 (1.8%) of 228 patients treated with moxifloxacin-based regimens had acquired fluoroquinolone resistance [16]. We found very low levels of acquired drug resistance in our study, irrespective of the fluoroquinolone used.

An obvious limitation of this analysis is the nature of the comparison between the two fluoroquinolones. Although all the data for this analysis were from the same clinical trial with the same inclusion criteria, assessments, and outcome measures, and the other drugs in the regimen were identical, inherent imbalances between the two groups are likely to be present due to changes over time in the population recruited. Randomised comparisons are well-balanced - the participants have similar characteristics between groups with respect to confounding variables, allowing the attribution of outcome differences to the study intervention with minimal bias, whereas in this analysis it is possible that some residual confounding remains. The sample size in this study is small and the comparison may, therefore, be underpowered to detect a difference between the fluoroquinolones if it truly exists. Strengths of the comparison are the high level of retention of the study population and the completeness of the data collected.

From the results of the present analysis, both from the weighted and adjusted analyses there is no significant difference between the two different fluoroquinolone containing regimens, although we note all analyses have point estimates that favour levofloxacin. This suggests that national treatment programmes can therefore select which fluoroquinolone they use based on other factors such as availability and other logistical constraints. However, our study is small and further work in this area is needed to confirm these findings.

## Figures and Tables

**Table 1:** Selected baseline characteristics (safety population)

		Levofloxacin N=62 (%)	Moxifloxacin N=65 (%)	p-value* (χ^2^-test)
Country	Ethiopia	2	19	
	Georgia	6	7	
	Moldova	17	8	-
	Mongolia	22	24	
	Uganda	15	7	
Sex	Male	38 (61%)	39 (60%)	0.88
	Female	24 (39%)	26 (40%)	
Age	< 25	13 (21%)	19 (29%)	
	25-44	33 (53%)	31 (64%)	0.56
	≥ 45	16 (26%)	15 (23%)	
Weight	< 50 kg	14 (23%)	24 (37%)	0.078
	≥ 50 kg	48 (77%)	41 (63%)	
HIV status	Negative	58 (94%)	60 (92%)	0.79
	Positive	4 (6%)	5 (8%)	
QTcF	< 400	19 (31%)	32 (49%)	0.033
	≥ 400	43 (69%)	33 (51%)	
Opacity	Minimal	9 (15%)	11 (18%)	
	Moderate	36 (59%)	36 (58%)	0.89
	Advanced	16 (26%)	15 (24%)	
	missing	1	3	
Cavitation	None	19 (31%)	14 (23%)	
	Single	13 (21%)	14 (23%)	0.55
	Multiple	29 (48%)	34 (55%)	
	missing	1	3	
Smear	No AFB, rare, 1+	23 (37%)	24 (37%)	
	2+	18 (29%)	9 (14%)	0.073
	3+	21 (34%)	32 (49%)	
Culture	Neg, MGIT, 1+	13 (21%)	15 (23%)	
	2+	24 (39%)	25 (38%)	0.96
	3+	25 (40%)	25 (38%)	

* Test for heterogeneity

**Table 2:** Efficacy analysis: Results of logistic regression, weighted and adjusted (mITT population)

Number Fav/Total	Analysis	Moxifloxacin OR^a^, 95%CI^b^ [p-value]	Difference, moxiflox-acin – levofloxacin, 95% CI [p-value]
87/115	Weighted	0.787 (0.322, 1.926) [0.60]	-0.045 (-0.213, 0.123) [0.60]
	Adjusted	0.526 (0.176, 1.571) [0.25]	-0.092 (-0.247, 0.063) [0.24]

a Odds Ratio

b Confidence Interval

**Table 3:** Safety analysis: Results of logistic regression, weighted and adjusted (safety population)

Safety outcome	Number Events/Total	Analysis	Moxifloxacin OR, 95%CI [p-value]	Difference, moxiflox-acin – levofloxacin, 95% CI [p-value]
Grade 3/4 AE	68/117	Weighted	1.478 (0.686, 3.185) [0.32]	0.091 (-0.087, 0.270) [0.32]
		Adjusted	1.626 (0.633, 4.174) [0.31]	0.109 (-0.098, 0.317) [0.30]
Grade 3/4 AE, excluding QT events	52/117	Weighted	1.055 (0.491, 2.263) [0.89]	0.013 (-0.177, 0.203) [0.89]
		Adjusted	1.410 (0.540, 3.677) [0.48]	0.074 (-0.129, 0.277) [0.48]
QTcF increase to ≥ 500 ms*	10/62	Weighted	1.715 (0.410, 7.174) [0.46]	0.083 (-0.136, 0.301) [0.46]
		Adjusted	1.671 (0.315, 8.856) [0.55]	0.069 (-0.150, 0.287) [0.54]
QTcF increase by 60 ms	39/118	Weighted	2.022 (0.878, 4.659) [0.098]	0.160 (-0.026, 0.346) [0.091]
		Adjusted	3.296 (0.993, 10.938) [0.051]	0.150 (0.006, 0.295) [0.042]

* analyses restricted to Mongolia and Uganda only as no events observed in other countries

## Data Availability

The original data presented in the study are available through TB-PACTS: https://c-path.org/tools-platforms/tb-pacts/ and TB-IPD: https://www.ucl.ac.uk/global-health/research/tb-ipd-platform
